# Fault Diagnosis for Complex Equipment Based on Belief Rule Base with Adaptive Nonlinear Membership Function

**DOI:** 10.3390/e25030442

**Published:** 2023-03-02

**Authors:** Zheng Lian, Zhijie Zhou, Xin Zhang, Zhichao Feng, Xiaoxia Han, Changhua Hu

**Affiliations:** 1 Missile Engineering Institute, PLA Rocket Force University of Engineering, Xi’an 710025, China; 2 College of War Support, PLA Rocket Force University of Engineering, Xi’an 710025, China

**Keywords:** fault diagnosis, belief rule base, membership function, model optimization

## Abstract

Fault diagnosis of complex equipment has become a hot field in recent years. Due to excellent uncertainty processing capability and small sample problem modeling capability, belief rule base (BRB) has been widely used in the fault diagnosis. However, previous BRB models almost did not consider the diverse distributions of observation data which may reduce diagnostic accuracy. In this paper, a new fault diagnosis model based on BRB is proposed. Considering that the previous triangular membership function cannot address the diverse distribution of observation data, a new nonlinear membership function is proposed to transform the input information. Then, since the model parameters initially determined by experts are inaccurate, a new parameter optimization model with the parameters of the nonlinear membership function is proposed and driven by the gradient descent method to prevent the expert knowledge from being destroyed. A fault diagnosis case of laser gyro is used to verify the validity of the proposed model. In the case study, the diagnosis accuracy of the new BRB-based fault diagnosis model reached 95.56%, which shows better fault diagnosis performance than other methods.

## 1. Introduction

With the rapid development of industrial technology, the fault diagnosis of complex equipment has received extensive attention and become a hot topic in Prognostics Health Management (PHM) [1,2,3]. At present, due to the complexity and intelligence of complex equipment in industrial production and national defense science and industry, it is tough to diagnose their faults through the appearance of the equipment. Therefore, establishing a fault diagnosis model is an effective method.

Current popular fault diagnosis models can be divided into three categories: mechanism-based models, knowledge-based models and data-driven models [4]. (1) Mechanism-based model: such models require that the mechanism of complex equipment can be clearly understood and corresponding mathematical or physical models can be established, such as the Kalman filter [5] and Kirchhoff law [6]. However, with the increasing complexity of equipment, its mechanism is difficult to grasp and such models are rarely used. (2) Knowledge-based model: this kind of model is established by the qualitative knowledge of domain experts, but it is generally difficult to achieve high modeling accuracy such as fault tree analysis (FTA) [7] and analytic hierarchy process (AHP) [8]. Since such models cannot learn by themselves, when the equipment is running, it cannot update knowledge and can only be modified by experts. (3) Data-driven model: with the advent of the big data era, data-driven fault diagnosis models have attracted a lot of attention. This kind of model does not need to master the mechanics of the equipment and can achieve modeling of the input and output relationship through the observation data. Simultaneously, they also have good learning abilities and can constantly be updated by data, such as with the support vector machines [9], decision trees [10] and deep learning algorithms [11]. However, in many fault diagnosis fields, the high-value fault samples are very limited, causing the data-driven model to fall into the problem of overfitting. On the other hand, due to the black-box nature of most data-driven models, the diagnosis results are not transparent and difficult to be convincing.

Belief rule base (BRB) is a generic rule-based modeling approach that was formally proposed by Yang et al. in 2006 [12]. It differs from traditional ML-type fuzzy rules: the consequent part of the “if-then” rule in BRB is composed of the belief distribution of all possible results, which means that BRB can handle fuzzy, uncertain and incomplete information together. Since rule-based knowledge representation is easy to understand by experts, BRB can embed expert knowledge easily and has good interpretability [13]. Therefore, as a knowledge-data hybrid-driven modeling method [14], BRB has been widely used in the field of fault diagnosis [15]. Xu et al. developed a fault diagnosis model of diesel engines based on BRB for the first time and realized the diagnosis of concurrent faults [16]. Feng et al. established a fault diagnosis model for oil pipelines based on BRB after considering the correlation of attributes [17]. Zhang et al. established the fault diagnosis and location model of the bus network based on BRB [18]. Li et al. developed an adaptive interpretable fault diagnosis model based on BRB [19]. Chen et al. proposed a mechanical equipment fault diagnosis model based on combination BRB [20]. Ming et al. proposed an interpretable fault diagnosis method based on probability table and BRB [21]. Wang et al. proposed a BRB-based fault diagnosis method for multi-agent systems [22].

As a typical fuzzy system, the first step of BRB in fault diagnosis is the transformation of input information. Since the observation information of most fault indicators in engineering are quantitative data, it needs to be fuzzified. In the previous BRB-based fault diagnosis model, the rule and utility based method is used to fuzzy quantitative data [23]: this is a triangular membership function [24]. The triangular membership function has a simple structure and can accurately transform the uniformly distributed observation data. However, in engineering, many observation data are not subject to the uniform distribution. Moreover, the small sample characteristics of fault data may present a distribution form that is difficult to be directly understood by people. In these cases, the triangular membership function is not accurate enough and will reduce the diagnostic accuracy. Therefore, it is necessary to adopt a membership function that can adapt the data distribution to transform the input information to improve the modeling accuracy.

As a commonly used membership function, the gaussian membership function can adapt to the distribution of data by adjusting its expectation and variance. Some research on this membership function in BRB has been carried out. Liu et al. introduced the Gaussian membership function in BRB-based engineering system safety analysis [25]; Zhang et al. also considered the Gaussian membership function when establishing the fault diagnosis model of the bus network [18]. However, it is worth noting that the adaptive ability of the Gaussian membership function is limited by the shape of the exponential curve. The Gaussian membership function is not ideal for the transformation of uniformly distributed observation data.

Therefore, a nonlinear membership function is proposed in this paper to make up for the shortcomings of the Gaussian membership function. In the exponential part of the original triangular membership function, a new parameter is considered to control the shape of the membership function curve. When the parameters change, the nonlinear membership function can adaptively transform the observation data under various distributions. Since the shape of the curve of the function changes more flexibly, the nonlinear membership function can more accurately convert the input information than the Gaussian membership function.

On the other hand, as an expert system, parameters of BRB model are initially determined by experts. Due to the inherent subjectivity and ignorance of experts, the diagnostic accuracy of the initial BRB is often not ideal and needs to be optimized. In recent years, many swarm intelligent algorithms have been developed for BRB parameter optimization, such as DE [26], PSO [27] and P-CMAES [28]. The common problem of these swarm intelligence algorithms is that expert knowledge is destroyed after optimization due to the random initialization of the population. This will cause BRB to lose its advantage in fault diagnosis. However, the gradient descent method searches from the parameters initially determined by experts, which allows retention of expert knowledge to the greatest extent. Therefore, a new optimization model based on the gradient descent method, which can further improve the modeling accuracy while maintaining expert knowledge, is developed. Therefore, the contributions of this paper are the following:

(1) A new BRB considering nonlinear membership function, which can adaptively deal with the non-uniform distribution of observation data in fault diagnosis, is proposed.

(2) For the new BRB model, a new optimization model based on the gradient descent method is proposed to improve the accuracy of fault diagnosis and keep expert knowledge from being destroyed.

The structure of this paper is as follows: In Section 2, the problem and basic knowledge are introduced. In Section 3, a new BRB-based fault diagnosis model with an adaptive membership function is proposed. In Section 4, a fault diagnosis case of the laser gyro is used to verify the validity of the proposed model. This paper is summarized in Section 5.

## 2. Problem Description and Basic Knowledge

### 2.1. Problem Description

Faced with the characteristics of few high-value fault samples and existing expert knowledge in the fault diagnosis for the complex equipment, this paper mainly solves the following three problems:

Problem 1: How to use a small amount of test data and existing expert knowledge to establish the fault diagnosis model. Due to the working characteristics of most complex equipment, the capacity of its observation data is very limited. Therefore, the data-driven fault diagnosis model is prone to overfitting since it cannot reflect all fault modes. On the other hand, in the long-term operation of the equipment, experts in the field have accumulated abundant expert knowledge that can be used to help judge the fault mode of the complex equipment. Therefore, the following mapping relationships need to be established:

(1) $$Y=R(x_{1},x_{2},\ldots ,x_{M},EK)$$

where $\left[x_{1},x_{2},\ldots ,x_{M}\right]$ represent M fault indicators. Y is the fault mode to be diagnosed. R(·) is the mapping function of the fault diagnosis model. EK is the expert knowledge.

Problem 2: For Problem 1, a belief rule-based fault diagnosis model will be proposed in Section 2.2. In engineering, observation data usually do not obey the uniform distribution. However, the triangular membership function in the previous BRB-based fault diagnosis model cannot accurately convert the non-uniformly distributed data, which will lead to poor fault diagnosis accuracy. Therefore, a new fuzzy membership function is needed to reflect the influence of uneven data on information transformation.

Problem 3: As an expert system, the parameters in the membership function of BRB are generally determined intuitively by experts according to domain knowledge and the distribution characteristics of observation data. However, due to the subjectivity and fuzziness of expert knowledge, these parameters cannot be given accurately, leading to a decrease in the modeling accuracy. Therefore, it is necessary to further improve the accuracy of fault diagnosis through parameter optimization.

### 2.2. Belief Rule-Based Fault Diagnosis Model

As a generalized ML-type fuzzy system, BRB is composed of a series of “if-then” belief rules, as shown below:

(2) $$\begin{array}{ll} R_{k}: & \mathrm{IF}\;x_{1}\;is\;H_{1}^{k}\;\wedge \;x_{2}\;is\;H_{2}^{k}\wedge \ldots \wedge x_{M}\;is\;H_{M}^{k},\;\\ & \mathrm{THEN}\;\left\{\left(D_{1},\beta_{1,k}\right),\left(D_{2},\beta_{2,k}\right)\ldots \left(D_{N},\beta_{N,k}\right)\right\},(\sum_{n=1}^{N}\beta_{n,k}\leq 1),\\ & \mathrm{with}\;a\;\mathrm{rule}\;\mathrm{weight}\;\theta_{k}(k=1,2\ldots ,L)\;\mathrm{and}\;\mathrm{attibute}\;\mathrm{weight}\;\delta_{i}(i=1,2\ldots ,T_{k}) \end{array}$$

where $\left[x_{1},x_{2},\ldots ,x_{M}\right]$ is the input vector and consists of m dimensions components. In fault diagnosis, $x_{i}$ is the ith fault indicator. $H_{i}^{k}$$\left(i=1,2,\ldots ,T_{k}\right)$ is the referential value of the $U_{i}(i=1,2,\ldots ,T_{k})$ attribute in the kth rule. It is generally determined by experts according to industry standards or observation data. L is the number of rules. $D_{n}(n=1,2,\ldots ,N)$ represents N possible faults. $\beta_{n,k}\;(n=1,2,\ldots ,N)$ represents the belief degree of the nth fault $D_{n}$, reflecting the support of the kth rule for this consequence. $\theta_{k}$ is the rule weight of the kth rule, which represents the relative importance of each rule. $\delta_{i}$ is the weight of the ith attribute, which reflects the relative importance of fault indicators.

Benefited from the knowledge representation based on the belief rule, BRB has the following advantages in fault diagnosis:

(1) Expert knowledge. Due to the natural advantages of language models, BRB uses rules to represent the nonlinear mapping relationship between fault indicators and fault modes, enabling experts and users to easily understand the behavior of the model. Therefore, compared with neural networks, support vector machines and decision tree models, the initial parameters of BRB can be determined by experts based on domain knowledge and existing observation data and the model is able to roughly reflect the mapping relationship of the system.

(2) Small sample modeling ability. The fault data of much equipment are characterized by high values and a small number of samples. Fortunately, due to the embedding of expert knowledge, BRB can model the system comprehensively with very limited observation data, even if the initial mapping relationship is rough. This means that BRB will not fall into the overfitting problem like the data-driven model. On the other hand, Chen et al. proved that BRB is a general approximation model [29], so this model has ideal modeling accuracy.

(3) Ability to process uncertain information. Compared with general fuzzy systems, BRB extends the “then” part of the rule to the belief distribution of all possible results, enabling BRB to deal with the probability uncertainty while solving the fuzzy uncertainty. Therefore, BRB can also show good performance when partial observation data are missing in the engineering application.

## 3. The Proposed Method

In this section, a BRB model with an adaptive nonlinear membership function is proposed to model the fault diagnosis considering non-uniform distribution observation data. In Section 3.1, the shortcomings of the existing triangular membership function and Gaussian membership function are analyzed in detail. Then, a nonlinear membership function is proposed to solve Problem 2. In Section 3.2, based on the gradient descent method, an optimization model considering the parameters of the nonlinear membership function is proposed to solve Problem 3.

### 3.1. Inference Process Based on the Nonlinear Membership Function

In fault diagnosis, all belief rules constitute a knowledge base. After the input information of the fault indicator is obtained, the fault mode can be diagnosed based on this knowledge base. It is worth noting that, as a fuzzy system, belief rules in BRB are expressed as mappings to linguistic values. However, the observation data of fault indicators are mostly quantitative information. Therefore, it is necessary to convert quantitative observation data into membership degree of all linguistic referential grades through a membership function, which is so-called “fuzzification” as follows:

(3) $$S(x_{i})=\{(H_{ij},\alpha_{ij});\;j=1,\ldots ,J_{i}\},\;\;\;\;i=1,2,\ldots M$$

where $H_{ij}$ is the jth referential grade of the ith fault indicator and $\alpha_{ij}$ is the corresponding membership degree. $x_{i}$ are the quantitative observation data.

In previous BRB models, the most commonly applied membership function is the triangular membership function, which is used in rule (or utility) based transformation methods, as shown below:

(4) $$\alpha_{ij}=\left\{\begin{array}{ll} \frac{a_{i(k+1)}-x_{i}}{a_{i(k+1)}-a_{ik}}, & j=k\;\mathrm{if}\;a_{ik}\leq A_{i}^{*}\leq a_{i(k+1)}\\ \frac{x_{i}-a_{ik}}{a_{i(k+1)}-a_{ik}}, & j=k+1\\ 0. & j=1,2,\ldots ,L,j\neq k,k+1 \end{array}\right.$$

where $\alpha_{ij}$ is a quantitative value corresponding to $H_{ij}$, which is usually determined by experts.

**Figure 1:**
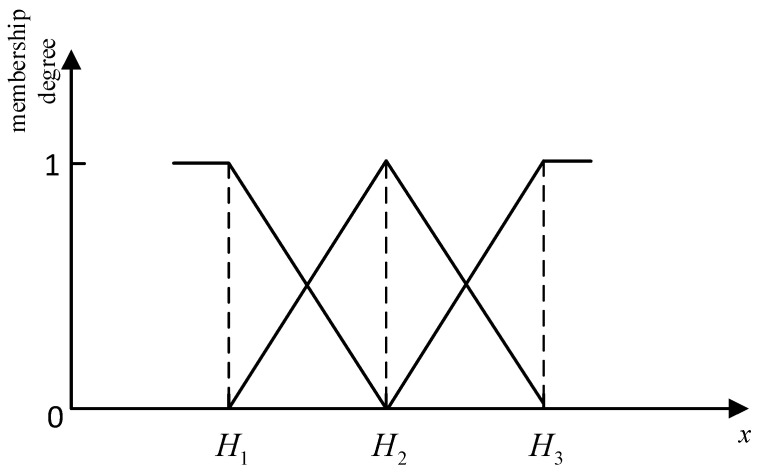
Membership degree using the triangular membership function.

**Figure 2:**
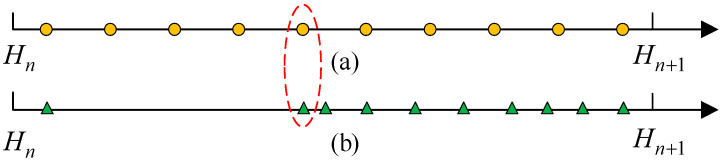
Comparison of uneven data distribution. The uniformly distributed data is shown in (**a**), and the non-uniformly distributed data is shown in (**b**).

The curve of the above membership function is shown in Figure 1. It can be seen from Figure 1 and Equation (4) that, since the derivative of this function is constant, the changing trend of membership of each referential grade is a straight line. However, when the observation data are uneven, such as when the data are concentrated in a certain area, this membership function cannot accurately reflect the corresponding membership, as shown in Figure 2. It can be seen from Figure 2a that the observation data are distributed evenly between the two referential grades. Therefore, it is easy to understand that the change in membership degree is linear in this case. But, in Figure 2b, the observation data are concentrated near the referential grade $H_{n+1}$. Therefore, for the two points marked by the red dotted line, their membership degree distribution should be different. The membership of the yellow point is assigned as $H_{n}=H_{n+1}=0.5$. For the green point in Figure 2b, since this point is closer to $H_{n}$ in the whole dataset, the membership degree of $H_{n}$ shall be greater than 0.5 and the membership degree of $H_{n+1}$ shall be less than 0.5, correspondingly.

For example, the two referential grades in Figure 2 correspond to the semantic values “low” and “high”, respectively. For the point marked in red in Figure 2b, it should be a lower value in the entire dataset. Therefore, the membership degree of the referential grade “low” should be higher.

The inaccurate quantitative data fuzzification will reduce the modeling accuracy of the fault diagnosis model. Thus, a nonlinear membership function is proposed for the fuzzification of uneven quantitative data in this paper, which can be described as follows:

(5) $$\alpha_{ij}=\left\{\begin{array}{ll} {\left(\frac{a_{i(k+1)}-x_{i}}{a_{i(k+1)}-a_{ik}}\right)}^{s}, & j=k\;\mathrm{if}\;a_{ik}\leq A_{i}^{*}\leq a_{i(k+1)}\;\\ 1-{\left(\frac{a_{i(k+1)}-x_{i}}{a_{i(k+1)}-a_{ik}}\right)}^{s}, & j=k+1\\ 0. & j=1,2,\ldots ,L,j\neq k,k+1 \end{array}\right.$$

where s∈(0,+∞) is the parameter of the function, which can reflect the distribution of observation data.

**Figure 3:**
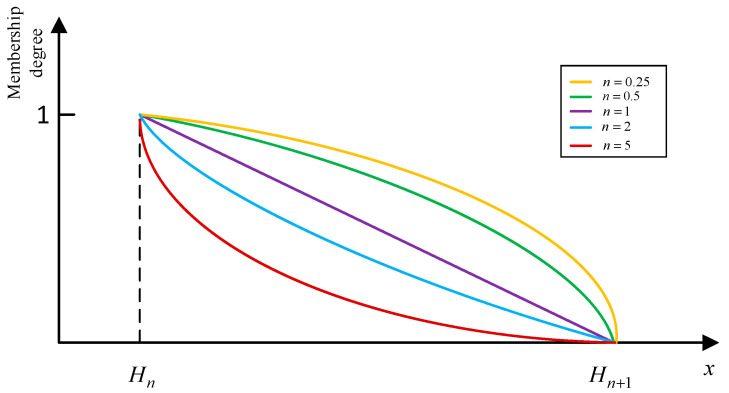
Membership degree of $H_{n}$ using the nonlinear membership function.

With the change of s, the new membership function can adaptively reflect the impact of different distributions of data. For example, when s is 0.25, 0.5, 1,2 and 5, respectively, the curves of the membership function are shown in Figure 3. It can be seen that with the increase of n, the function changes from convex to concave. In particular, when s equals 1, the nonlinear membership function degenerates into a triangular membership function. Correspondingly, for the distribution of data in Figure 2b, the nonlinear membership function at s= 0.25 or 0.5 can more accurately conduct the fuzzification of quantitative data in this case.

It is worth noting that, as another commonly used membership function in fuzzy systems, the Gaussian membership function can also realize adaptive fuzzification of input data through changes in expectation and standard deviation, as shown below:

(6) $$\alpha_{ij}=\exp\left(-\frac{1}{2}{\left(\frac{x_{i}-c_{ij}}{\sigma_{ij}}\right)}^{2}\right)$$

where $c_{ij}$ is the expectation and $\sigma_{ij}$ is the standard deviation.

**Figure 4:**
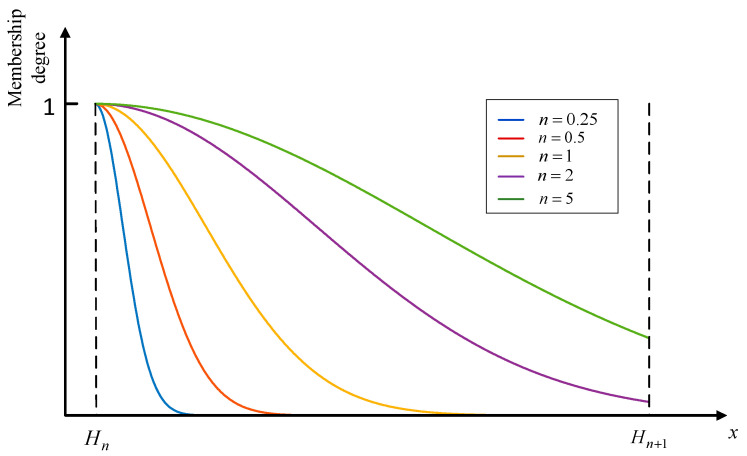
Membership degree of $H_{n}$ using the Gaussian membership function.

However, it has the following two shortcomings: firstly, the Gaussian membership function cannot achieve accurate transformation of uniformly distributed input information, which is limited by the characteristics of its nonlinear curve. However, when s=1, the nonlinear membership function proposed in this paper can avoid this problem. Secondly, the adaptive ability of the Gaussian membership function is insufficient. For the data distribution within an interval, the Gaussian membership function can only self-adapt the data distribution under partial circumstances, as shown in Figure 4. The standard variance of the Gaussian membership function is 0.25, 0.5, 1, 2 and 5, respectively, in Figure 4. With the increase of variance, the shape of the exponential function curve cannot properly reflect the distribution characteristics of the dataset close to $H_{n+1}$. Furthermore, when the input is at $H_{n+1}$, the membership degree of $H_{n}$ is still quite high, which is difficult for users to understand. Therefore, the Gaussian membership function is only applicable when the observation data are concentrated near the referential grade $H_{n}$. Based on the above analysis, it can be seen that the nonlinear membership function proposed in this paper can more accurately reflect the distributions of data.

Therefore, based on the nonlinear membership function, when observation data are obtained, the steps for fault diagnosis can be described as follows:

**Step 1:** Fuzzification of quantitative data. The referential grade of each fault indicator is a fuzzy partition, which is assigned to the nonlinear membership function $R{\left(\cdot \right)}_{ij}$. For the observation data of ith fault indicator, the membership degree of each referential grade is calculated as follows:

(7) $$\alpha_{ij}=R_{ij}(x_{i})=\left\{\begin{array}{ll} {\left(\frac{a_{i(k+1)}-x_{i}}{a_{i(k+1)}-a_{ik}}\right)}^{s_{ij}}, & j=k\;\mathrm{if}\;a_{ik}\leq x_{i}\leq a_{i(k+1)}\\ 1-{\left(\frac{a_{i(k+1)}-x_{i}}{a_{i(k+1)}-a_{ik}}\right)}^{s_{ij}}, & j=k+1\\ 0. & j=1,2,\ldots ,L,j\neq k,k+1 \end{array}\right.$$

where $x_{i}$ are the input data. $s_{ij}$ is the parameter of the nonlinear membership function, which is usually determined by experts after observing the distribution of data or calculated based on statistical methods.

**Step 2:** Activation of belief rules. The activation weight of the rule is calculated as follows:

(8) $$w_{k}=\frac{\theta_{k}\alpha_{k}}{\overset{L}{\underset{l=1}{\sum }}\theta_{l}\alpha_{l}}$$

where

(9) $$\alpha_{k}=\overset{M_{k}}{\underset{i=1}{\prod }}{\left(\alpha_{i}^{k}\right)}^{\bar{\delta_{i}}},\bar{\delta_{i}}=\frac{\delta_{i}}{\underset{i=1,2,\ldots M_{k}}{\max}\{\delta_{i}\}}$$

**Step 3:** Reasoning of activated rules. In this paper, the analytic ER algorithm [30] is used to fuse the activated rules to obtain the belief degree of each failure mode as follows:

(10) $$\begin{array}{l} {\hat{\beta }}_{n}=\frac{\mu [\prod_{k=1}^{L}\left(w_{k}\beta_{n,k}+1-w_{k}\sum_{j=1}^{N}\beta_{j,k}\right)-\prod_{k=1}^{L}\left(1-w_{k}\sum_{j=1}^{N}\beta_{j,k}\right)]}{1-\mu [\prod_{k=1}^{L}\left(1-w_{k}\right)]},\\ n=1,2,\ldots N,\\ \mu =[\sum_{n=1}^{N}\prod_{k=1}^{L}\left(w_{k}\beta_{n,k}+1-w_{k}\sum_{j=1}^{N}\beta_{j,k}\right)-(N-1)\prod_{k=1}^{L}\left(1-w_{k}\sum_{j=1}^{N}\beta_{j,k}\right){]}^{-1} \end{array}$$

where ${\hat{\beta }}_{n}$ represents the belief degree of the nth failure mode $D_{n}$.

In general, the failure mode with the highest belief degree is regarded as a possible failure as the output of the model as follows:

(11) $$\hat{n}=\underset{n}{\arg\max}({\hat{\beta }}_{n})$$

where $\hat{n}$ indicates the diagnosed fault mode.

### 3.2. Model Optimization Based on the Gradient Descent Method

Due to the subjectivity and fuzziness of expert knowledge, the modeling accuracy of the initially constructed fault diagnosis model is generally difficult to meet the requirements of practical engineering. Therefore, the model parameters initially determined by experts in the BRB need to be optimized to improve the diagnostic accuracy of the model. In general, for classification problems such as fault diagnosis, the cross-entropy loss function is used as the objective function as follows:

(12) $$Q(\Omega )=-\frac{1}{T}\sum_{t=1}^{T}\sum_{j=1}^{N}y_{j}^{t}\log_{2}{\hat{\beta }}_{j}^{t},y_{j}^{t}=\left\{\begin{array}{c} 0\left(j\neq {\hat{y}}_{t}\right)\\ 1\left(j={\hat{y}}_{t}\right) \end{array}\right.$$

where $\hat{y}\in \{1,2\ldots ,N\}$ indicates the category of the real fault. T is the capacity of observation data. Ω={θ,δ,β,s} is a parameter vector, which is composed of rule weight, attribute weight, basic belief degree and parameters of the membership function.

Considering the constraint conditions of parameters in BRB model, the following parameter optimization model can be constructed:

(13) $$\begin{array}{l} \min\;Q(\{\theta ,\delta ,\beta ,s\})=-\frac{1}{T}\overset{T}{\underset{t=1}{\sum }}\overset{N}{\underset{j=1}{\sum }}y_{j}^{t}\log_{2}{\hat{\beta }}_{j}^{t},y_{j}^{t}=\left\{\begin{array}{c} 0\left(j\neq {\hat{y}}_{t}\right)\\ 1\left(j={\hat{y}}_{t}\right) \end{array}\right.\\ s.t.\\ 0\leq \theta_{k}\leq 1,0\leq \delta_{i}\leq 1,0\leq \beta_{n,k}\leq 1,\overset{N}{\underset{n=1}{\sum }}\beta_{n,k}\leq 1,s_{ij}>0\\ \left(k=1,2,\ldots ,L,i=1,2,\ldots ,M,n=1,2,\ldots ,N,j=1,2,\ldots ,J_{i}\right) \end{array}$$

In recent years, many optimization algorithms have been developed for BRB model parameter optimization, such as DE, PSO, P-CMAES and other swarm intelligence algorithms. Yang et al. [31] pointed out that when BRB is used as an expert system, the optimization of model parameters should only be “fine-tuning”, which is also a major difference between BRB and artificial neural networks. Feng et al. [32] pointed out that due to the operation of population initialization of swarm intelligence algorithm, the expert knowledge in BRB is likely to be destroyed and the reasoning results may conflict with intuition. This may cause the fault diagnosis results to be difficult to be convincing and weaken the interpretability of the model. Compared with the swarm intelligence algorithm, the gradient descent method directly uses derivative information and takes the parameters initially determined by experts as the initial value of optimization to search, retaining initial expert knowledge to the greatest extent. Therefore, in this paper, stemming from the derivability of the BRB reasoning process, an optimization algorithm based on gradient descent is used to train the model.

There are 4 types of parameters as optimization variables. Therefore, it is necessary to calculate the first-order partial derivative of the objective function with respect to them.

First, the first-order partial derivative of the objective function Q with respect to the reasoning result ${\hat{\beta }}_{n}$ is calculated as follows:

(14) $$\frac{\partial Q}{\partial {\hat{\beta }}_{n}}=-\frac{1}{T}\overset{T}{\underset{t=1}{\sum }}y_{j}^{t}\frac{1}{{\hat{\beta }}_{n}^{t}},y_{j}^{t}=\left\{\begin{array}{c} 0\left(j\neq {\hat{y}}_{t}\right)\\ 1\left(j={\hat{y}}_{t}\right) \end{array}\right.$$

The first-order partial derivative of the reasoning result ${\hat{\beta }}_{n}$ with respect to the basic belief degree $\beta_{r,f}$ is:

(15) $$\frac{\partial {\hat{\beta }}_{n}}{\partial \beta_{r,f}}=\left\{\begin{array}{c} {\left.\frac{\left[\overset{N}{\underset{p=1}{\sum }}\xi (p)-(N-1)\overset{L}{\underset{k=1}{\prod }}\left(1-w_{k}\right)\right]\xi (n)\frac{w_{f}}{w_{f}\beta_{r,f}+1-w_{f}}}{\left[\overset{N}{\underset{p=1}{\sum }}\xi (p)-N\right.}\overset{L}{\underset{k=1}{\prod }}\left(1-w_{k}\right)\right]}^{2}\\ \frac{\left[\overset{L}{\underset{k=1}{\prod }}\left(1-w_{k}\right)-\xi (n)\right]\xi (r)\frac{w_{f}}{w_{f}\beta_{r,f}+1-w_{f}}}{{\left[\overset{N}{\underset{p=1}{\sum }}\xi (p)-N\overset{L}{\underset{k=1}{\prod }}\left(1-w_{k}\right)\right]}^{2}},\;r\neq n \end{array}\right.$$

where

(16) $$\xi (p)=\overset{L}{\underset{k=1}{\prod }}\left(w_{k}\beta_{p,k}+1-w_{k}\right)$$

So far, the first-order partial derivative of the first type of parameter has been calculated as follows:

(17) $$\frac{\partial Q}{\partial \beta_{r,k}}=\overset{N}{\underset{n=1}{\sum }}\frac{\partial Q}{\partial {\hat{\beta }}_{n}}\frac{\partial {\hat{\beta }}_{n}}{\partial {\hat{\beta }}_{r,k}}$$

Then, we need to calculate the first-order partial derivative of rule weight, attribute weight and parameters of the membership function. According to the chain rule, the first-order partial derivative of the reasoning result ${\hat{\beta }}_{n}$ with respect to the activation weight $w_{g}$ needs to be obtained as follows:

(18) $$\begin{array}{l} \frac{\partial {\hat{\beta }}_{n}}{\partial w_{f}}=\frac{[A(n)C(n)\sum_{p=1}^{N}A(p)-A(n)\sum_{p=1}^{N}A(p)C(p)-NBA(n)C(n)-}{{\left[\sum_{p=1}^{N}A(p)-NB\right]}^{2}}\\ =\frac{\prod_{\begin{array}{l} k=1\\ k\neq f \end{array}}^{L}\left(1-w_{k}\right)\sum_{p=1}^{N}A(p)+NA(n)\prod_{\begin{array}{l} k=1\\ k\neq f \end{array}}^{L}\left(1-w_{k}\right)+B\sum_{p=1}^{N}A(p)C(p)]}{{\left[\sum_{p=1}^{N}A(p)-NB\right]}^{2}} \end{array}$$

where

(19) $$\begin{array}{l} A(n)=\overset{L}{\underset{k=1}{\prod }}\left(w_{k}\beta_{n,k}+1-w_{k}\right)\\ B=\overset{L}{\underset{k=1}{\prod }}\left(1-w_{k}\right)\\ C(n)=\frac{\beta_{n,t}-1}{w_{t}\beta_{n,t}+1-w_{t}} \end{array}$$

The first derivative of the activation weight $w_{t}$ with respect to the rule weight $\theta_{f}$ is calculated as follows:

(20) $$\frac{\partial w_{t}}{\partial \theta_{f}}=\left\{\begin{array}{l} \frac{\sum_{l=1,l\neq t}^{L}\theta_{l}\alpha_{l}\alpha_{f}}{(\sum_{l=1}^{L}\theta_{l}\alpha_{l}{)}^{2}},t=f\\ -\frac{\theta_{t}\alpha_{t}\alpha_{f}}{(\sum_{l=1}^{L}\theta_{l}\alpha_{l}{)}^{2}},t\neq f \end{array}\right.$$

For the attribute weight $\delta_{i}$, the normalization of this parameter in Equation (9) is nondifferentiable. Therefore, only the first order partial derivative of the normalized attribute weight ${\bar{\delta }}_{i}$ can be calculated here. First, the first derivative of the activation weight $w_{t}$ with respect to the rule matching degree $\alpha_{f}$ needs to be calculated:

(21) $$\frac{\partial w_{t}}{\partial \alpha_{f}}=\left\{\begin{array}{l} \frac{\sum_{l=1,l\neq t}^{L}\theta_{l}\alpha_{l}\theta_{f}}{(\sum_{l=1}^{L}\theta_{l}\alpha_{l}{)}^{2}},t=f\\ -\frac{\theta_{t}\alpha_{t}\theta_{f}}{(\sum_{l=1}^{L}\theta_{l}\alpha_{l}{)}^{2}},t\neq f \end{array}\right.$$

Then, the first derivative of rule matching degree $\alpha_{f}$ with respect to normalized attribute weight ${\bar{\delta }}_{{}_{i}}$ is calculated as follows:

(22) $$\frac{\partial \alpha_{f}}{\partial {\bar{\delta }}_{{}_{i}}}=\alpha_{f}\log(\alpha_{i}^{k})$$

Therefore, according to the chain rule, the partial derivative of the objective function with respect to the rule weight and the normalized attribute weight can be calculated as follows:

(23) $$\frac{\partial Q}{\partial \theta_{k}}=\overset{L}{\underset{t=1}{\sum }}\overset{N}{\underset{n=1}{\sum }}\frac{\partial Q}{\partial {\hat{\beta }}_{n}}\frac{\partial {\hat{\beta }}_{n}}{\partial w_{t}}\frac{\partial w_{t}}{\partial \theta_{k}}$$

(24) $$\frac{\partial Q}{\partial {\bar{\delta }}_{i}}=\overset{L}{\underset{k=1}{\sum }}\overset{L}{\underset{t=1}{\sum }}\overset{N}{\underset{n=1}{\sum }}\frac{\partial Q}{\partial {\hat{\beta }}_{n}}\frac{\partial {\hat{\beta }}_{n}}{\partial w_{t}}\frac{\partial w_{t}}{\partial \alpha_{k}}\frac{\partial \alpha_{k}}{\partial {\bar{\delta }}_{i}}$$

Finally, the first order partial derivative of the individual membership degree $\alpha_{i}^{k}$ with respect to the parameters of the membership degree function $s_{ij}$ is calculated as follows:

(25) $$\frac{\partial \alpha_{ij}}{\partial s_{ij}}=\left\{\begin{array}{l} {\left(\frac{a_{i(k+1)}-x_{i}}{a_{i(k+1)}-a_{ik}}\right)}^{s_{ij}}\log(\frac{a_{i(k+1)}-x_{i}}{a_{i(k+1)}-a_{ik}}),\;\;\;\;\;\;\;\;j=k\;\mathrm{if}\;a_{ik}\leq x_{i}\leq a_{i(k+1)}\\ -{\left(\frac{a_{i(k+1)}-x_{i}}{a_{i(k+1)}-a_{ik}}\right)}^{s_{ij}}\log(\frac{a_{i(k+1)}-x_{i}}{a_{i(k+1)}-a_{ik}}),\;\;\;\;j=k+1\\ 0.\;\;\;\;\;\;\;\;\;\;\;\;\;\;\;\;\;\;\;\;\;\;j=1,2,\ldots ,L,j\neq k,k+1 \end{array}\right.$$

According to the chain rule, the partial derivative of the objective function with respect to the parameters of the membership function is calculated as follows:

(26) $$\frac{\partial Q}{\partial s_{ij}}=\sum_{k=1}^{L}\sum_{t=1}^{L}\sum_{n=1}^{N}\frac{\partial Q}{\partial {\hat{\beta }}_{n}}\frac{\partial {\hat{\beta }}_{n}}{\partial w_{t}}\frac{\partial w_{t}}{\partial \alpha_{k}}\frac{\partial \alpha_{k}}{\partial \alpha_{i}^{k}}\frac{\partial \alpha_{i}^{k}}{\partial s_{ij}}$$

Therefore, the gradient vector of the optimization variable can be obtained as follows:

(27) $$d={\left[\frac{\partial Q}{\partial \beta_{n,k}}\;\frac{\partial Q}{\partial \theta_{k}}\;\frac{\partial Q}{\partial {\bar{\delta }}_{i}}\;\frac{\partial Q}{\partial s_{ij}}\right]}^{T}$$

Since each parameter in the optimization model has corresponding constraints, they should be approximated to meet the optimization constraints after the parameter is updated based on the gradient. Thus, the steps of parameter optimization can be summarized as follows:

**Step 1:** The model parameters initially given by experts: basic belief degree, rule weight, attribute weight and parameters of membership function are taken as the initial value $z_{k}=z_{0}$.

**Step 2:** Calculate the gradient of the optimization variable $d_{k}$.

**Step 3:** The optimization variables are updated as follows:

(28) $$z_{k+1}=z_{k}-\lambda d_{k}$$

where λ is the step size of iteration and is determined by the one-dimensional search method.

**Step 4:** Approximate projection operation. For inequality constraints of each parameter, when the value of the parameter does not meet the constraint conditions, take the adjacent bound as the approximate value. For example, if $\theta_{k+1}<0$, then $\theta_{k+1}^{\prime }=0$. Moreover, the basic belief degree of a belief rule is normalized so that the sum is 1. Therefore, $z_{k+1}^{\prime }$ is obtained.

**Step 5:** Calculate the gradient vector $d_{k+1}$ at this time. Judge whether the termination condition of the algorithm is reached. If yes, end. Otherwise, let $d_{k}=d_{k+1},z_{k}=z_{k+1}^{\prime }$ and go to Step 3.

**Figure 5:**
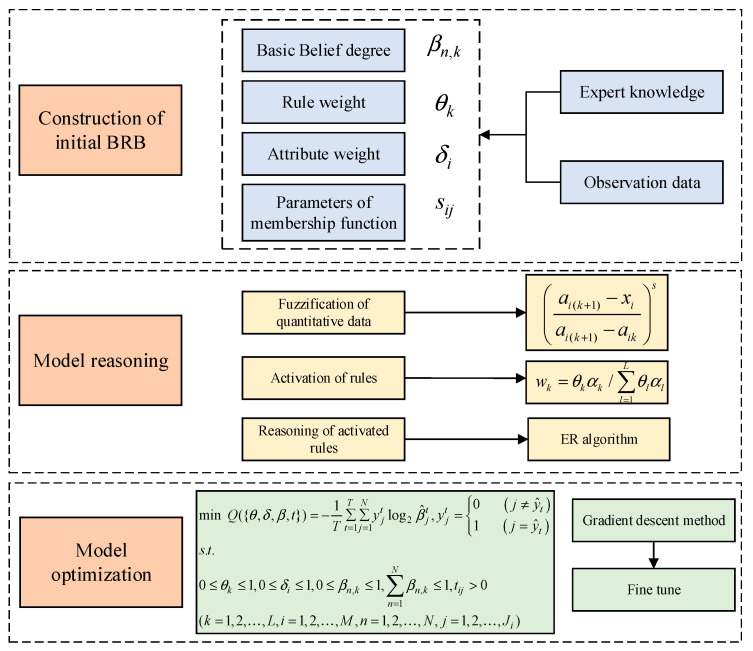
The whole process of the proposed model.

Finally, the fault diagnosis model proposed in this paper can be summarized as shown in Figure 5.

## 4. Case Study

A fault diagnosis case of a laser gyro will be used in this section to verify the effectiveness of the proposed model. In Section 4.1, the background of laser gyro fault diagnosis is briefly introduced. In Section 4.2, the BRB-based fault diagnosis model is built and optimized. In Section 4.3, a comparative study between the proposed model and other models is conducted. Analysis and discussions are carried out in Section 4.4.

### 4.1. Background Description

**Figure 6:**
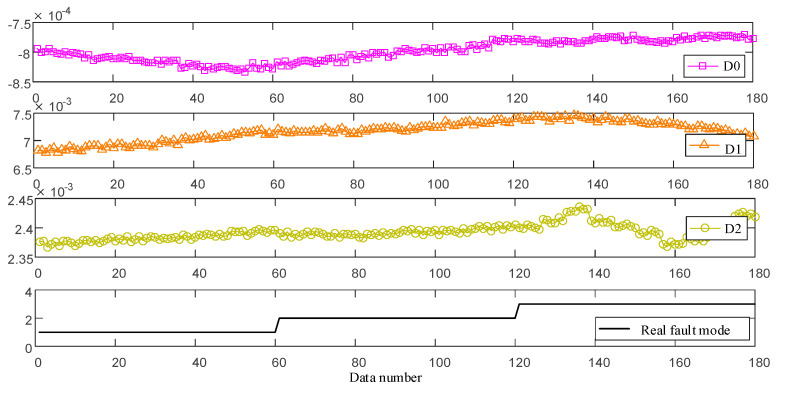
Observation data of fault indicators.

As an important navigation device, the laser gyro plays an extremely important role in many fields, such as automobiles, ships, rockets, etc. However, in the storage process of the laser gyro, due to the inevitable external interference and its own performance degradation, it is very likely to be in the fault state. Once these laser gyros are used in the failure state, it may cause unbearable personnel and property losses. For the laser gyro, it is difficult to judge whether it is in the fault state from appearance. However, the observation data of its drift coefficient can reflect the degree of the fault. In general, the greater the drift coefficient, the higher the degree of the fault. Therefore, in this paper, the zero-order term drift coefficient $D_{0}$, the first-order term drift coefficient $D_{1}$ and the second-order term drift coefficient $D_{2}$ are used as indicators of laser gyro fault diagnosis. For a certain laser gyro, 180 groups of observation data within a storage period are shown in Figure 6. According to the fault degree of laser gyro and the industry standard, the three fault modes are, respectively, slight fault (S), moderate fault (M) and bad fault (B). This is shown in Figure 6.

### 4.2. Construction and Optimization of the Fault Diagnosis Model

In this case study, the three drift coefficients of the laser gyro are fault indicators which are used to diagnose three types of fault modes, namely slight fault (S), moderate fault (M) and bad fault (B). Therefore, the following BRB can be established:

$$R_{k}:\;\mathrm{IF}\;D_{0}\;is\;H_{1}^{k}\;\wedge \;D_{1}\;is\;H_{2}^{k}\wedge D_{2}\;is\;H_{3}^{k},\mathrm{THEN}\;\left\{\left(B,\beta_{1,k}\right),\left(M,\beta_{2,k}\right),\left(S,\beta_{3,k}\right)\right\}$$

According to expert knowledge and industry standards, each fault indicator has three referential grades, namely low (L), medium (M) and high (H), whose corresponding referential values are shown in Table 1. Thus, the “then” part of the rules in the initial BRB is shown in Table 2. All rule weights are initially set to one. Since $D_{0}$ can most obviously reflect the degree of fault and $D_{1}$ takes the second place, the attribute weights are set to $\delta_{1}=1,\delta_{2}=0.7$ and $\delta_{3}=0.5$. According to the distribution of the observation data, the initial parameters of the nonlinear membership function are shown in Table 3.

**Figure 7:**
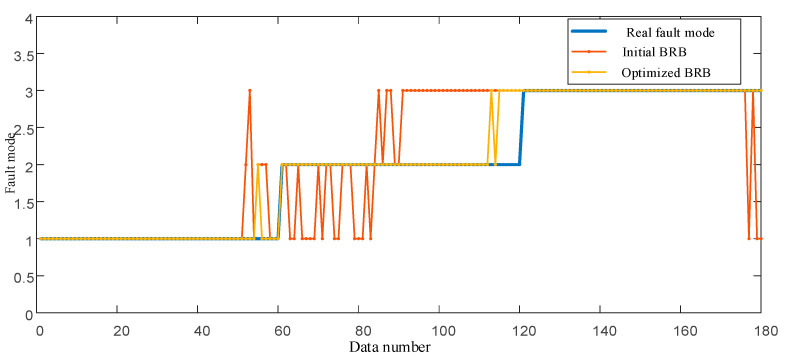
Comparison between initial BRB and optimized BRB.

Since it is difficult for the initially constructed BRB to achieve ideal modeling accuracy, observation data are required to optimize the model. For 180 groups of observation data, 30% are randomly selected as the training set and the rest as the test set to reflect the modeling ability of BRB in small sample problems. The gradient descent method in Section 4.3 is used as the optimization engine. The optimized model parameters are shown in Table 4. In addition, optimized parameters of the nonlinear membership function are shown in Table 5. The fault diagnosis results of optimized BRB and initial BRB are shown in Figure 7.

### 4.3. Comparative Study

In order to fully verify the effectiveness of the model in this paper, comparative experiments are carried out in this section from the following two aspects, namely, the previous BRB model and data-driven models.

- The previous BRB model

(1) The BRB model with triangular membership function, named BRB-t: the input information transformation function of this BRB adopts the triangular membership function in Equation (4). BRB-tri also uses the gradient descent method to optimize model parameters.

(2) The BRB model with Gaussian membership function, is named BRB-g: the optimization method of this model is the same as BRB-t.

**Figure 8:**
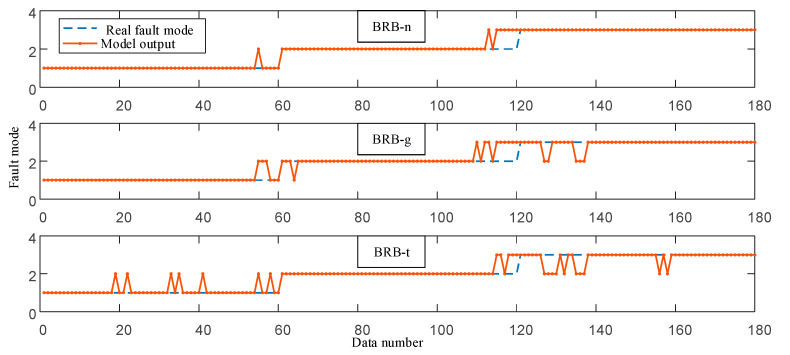
Comparison of diagnosis results of different BRBs.

Correspondingly, the BRB proposed in this paper is named BRB-n. The fault diagnosis results of three BRBs on 180 sets of observation data are shown in Figure 8. The accuracy of test data is shown in Table 6.

b. Data-driven models

Data-driven fault diagnosis methods have been widely used. In this paper, Random forest (RF), Naive Bayes (NB) and K-nearest neighbor (KNN) models are used for comparative study.

(1) RF: RF is a type of powerful tree ensemble model [33]. Its basic model is the decision tree (DT). Compared with general DT, RF has a stronger generalization ability, so it is widely used in classification and regression problems.

(2) NB: NB is a nonparametric model based on the Bayesian theorem [34]. This model has no explicit learning process. Generally, it calculates the prior probability and the conditional probability directly from the training set and infers a posteriori probability.

(3) KNN: KNN is a lazy machine learning model [35], which means that it has no model training process. For the data to be predicted, the training data closest to this data are first obtained according to the defined distance formula. Then, their weighted averages or votes are calculated.

**Figure 9:**
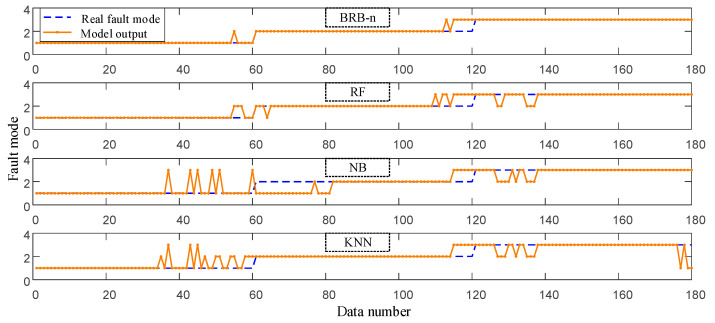
Comparison of diagnosis results of d data-driven models.

The hyper-parameters of these models are the default settings in the Python “sklearn” library. They are then adjusted by the “GridSearchCV” function. Their diagnostic results and accuracy are shown in Figure 9 and Table 7, respectively.

c. Swarm intelligence algorithms

To illustrate the advantages of the gradient-based optimization algorithm proposed in this paper, three swarm intelligence algorithms, that is, DE, PSO and P-CMAES, are used to optimize the initial BRB model. Their parameter settings are the same as those in [23,24,25]. The optimized BRBs are named BRB-DE, BRB-PSO and BRB-PCMAES, respectively. For comparison, the BRB optimized by the proposed method is named BRB-GB. The accuracy of fault diagnosis of these models is shown in Table 8.

### 4.4. Analysis and Discussions

**Figure 10:**
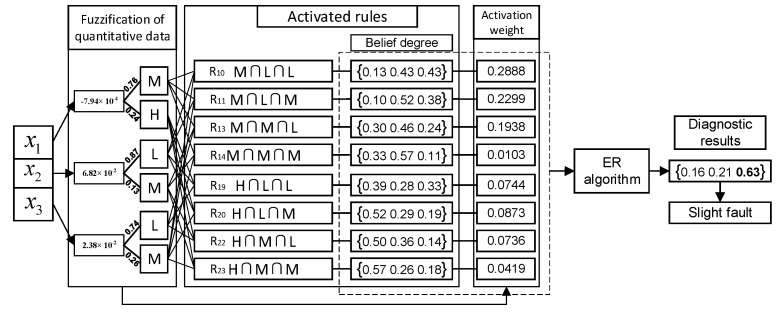
Visualization of fault diagnosis based on BRB.

Firstly, the advantages of the BRB-based fault diagnosis model are described by comparing it with data-driven models. In this paper, 30% of samples of the dataset are selected as the training set to train a wide variety of fault diagnosis models. Under this circumstance, the drawbacks of the data-driven model are revealed. Since the training set may not comprehensively reflect the overall mapping relationship, these models almost fall into the problem of overfitting. Among them, with the advantage of a double random sampling of features and samples, the random forest model alleviates the problem to some extent and has the highest diagnostic accuracy among several data-driven models. By comparing Table 5 and Table 6, it can be seen that after model optimization, the fault diagnosis accuracy of several BRBs are higher than that of data-driven models. Among them, the diagnostic accuracy of BRB-n has been improved by 12.8%, 25.24% and 31.07%, respectively. This reveals the advantage of expert knowledge in small sample modeling; that is, experts construct a generally correct but rough model by virtue of domain knowledge and experience. Then, the diagnosis accuracy of the model is further improved through the existing small sample dataset. On the other hand, as a “white-box” model, the BRB model provides explicit knowledge representation and reasoning compared with data-driven methods, enabling the results of fault diagnosis to be traceable and transparent. In order to further illustrate the interpretability of BRB, the fault diagnosis process of the first observation data [−7.94 × 10^−4^ 6.82 × 10^−2^ 2.38 × 10^−2^] is shown in Figure 10.

Secondly, the advantages of the gradient-based optimization method are analyzed. On the one hand, the gradient-based optimization algorithm shows powerful model training ability. Compared with the other three swarm intelligence algorithms, the modeling accuracy of BRB-GB has improved by 7.87%, 6.44% and 2.95%, respectively. On the other hand, the proposed optimization algorithm can keep expert knowledge from being destroyed, which is reflected in that the optimized model parameters will not be significantly changed, but rather, fine-tuned. For simplicity, the belief degrees of the first rule in four BRBs are shown in Table 9. Table 9 shows that, due to random initialization, the distribution of belief degree of the BRB optimized by the swarm intelligence algorithm has seriously deviated from the initial judgment of experts, even if they can achieve good fault diagnosis accuracy. This will make the rules difficult to understand; when the referential value of the evaluation indicator is low, the fault degree is generally “slight” according to common sense and experience. When these BRBs are used for fault diagnosis, the interpretability of the diagnosis results will be weakened.

Finally, the importance of the nonlinear membership function is analyzed. First, the uneven distribution of observation data will affect the accuracy of the fault diagnosis model. It can be seen from Table 6 that the diagnosis accuracy of BRB is improved after considering the adaptive membership function. This is easy to understand since the original triangular membership function cannot reflect the diversity of different data distributions, leading to errors in the fuzzification of input information. Therefore, it is necessary to consider the adaptive membership function when building a fault diagnosis model based on BRB. Then, compared with the other two BRBs, the diagnostic accuracy of BRB-n is increased by 5.31% and 9.57%, respectively. It is worth noting that the Gaussian membership function has more adjustment parameters than the nonlinear membership function. For BRB-g and BRB-n in this paper, the optimization parameters are 123 and 117, respectively. With the increase of the number of the referential grade, the difference in the number of parameters will continue to expand. However, the nonlinear membership function can show better performance. This is because, compared with the Gaussian membership function, the nonlinear membership function can adapt to a wider range of data distributions and can more precisely conduct the fuzzification of quantitative data.

**Figure 11:**
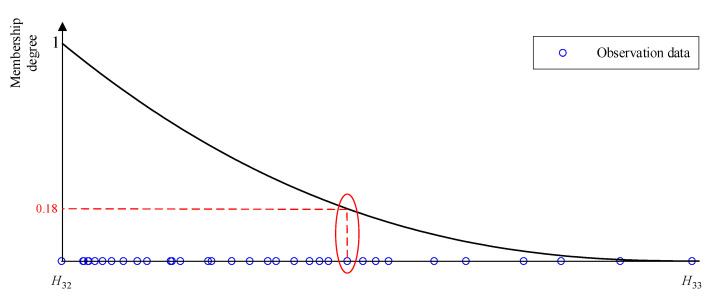
Membership degree of data with non-uniform distribution.

In order to further verify the role of information transformation of nonlinear membership function, part of the observation data of $D_{2}$ is shown in Figure 11. The observation data are concentrated on $H_{32}$ in the interval $\left[a_{32},a_{33}\right]$. Therefore, in the entire dataset, the red-marked areas are more subordinate in $H_{33}$. In other words, this point should belong to the referential grade “high” in the entire dataset to a greater extent. According to the optimized BRB reasoning process, the membership degrees of $H_{32}$ and $H_{33}$ are 0.18 and 0.82, respectively, which is consistent with the real distribution of the dataset. Therefore, when the nonlinear membership function is used to transform the input information, BRB can achieve a more ideal modeling accuracy.

## 5. Conclusions

In this paper, a new fault diagnosis model based on BRB has been proposed. In order to address the distribution of different data, an adaptive nonlinear membership function has been proposed to conduct the fuzzification of quantitative data. Since the parameters of the membership function initially determined by experts may not be accurate in the new BRB model, a new parameter optimization model considering the parameters of the membership function has been proposed with the aid of by the gradient descent algorithm. Finally, the proposed model is verified by a laser gyro fault diagnosis case.

In summary, the proposed method has two advantages: firstly, in the transformation of input information, the limitations of the triangular membership function in the fuzzification of non-uniformly distributed observation data are considered for the first time and an adaptive nonlinear membership function is designed. This function can adapt to the distribution of various data and improve the accuracy of information transformation. Secondly, considering the subjectivity and ignorance of experts in determining the parameters of the model, the parameters of the membership function are added to the optimization model; the gradient descent method is used to optimize the fault diagnosis model, enabling expert knowledge to not be destroyed and improving modeling accuracy.

Furthermore, the model optimization of BRB is a non-convex optimization problem, which means that the traditional gradient descent method may fall into the local optimal value. Therefore, it is interesting to get higher modeling accuracy by jumping out of the local optimal value. This issue will be considered in the future.

## Figures and Tables

**Table 1:** Referential values of fault indicators.

	L	M	H
$D_{0}$	−8.32 × 10^−4^	−8.01 × 10^−4^	−7.69 × 10^−4^
$D_{1}$	6.8 × 10^−2^	7.1 × 10^−2^	7.5 × 10^−2^
$D_{2}$	2.36 × 10^−2^	2.4 × 10^−2^	2.43 × 10^−2^

**Table 2:** The rules of initial BRB.

No.	$\theta_{l}$	$D_{0}\wedge D_{1}\wedge D_{2}$	Consequent {B,M,S}	No.	$\theta_{l}$	$D_{0}\wedge D_{1}\wedge D_{2}$	Consequent {B,M,S}
1	1	L∧L∧L	{0 0 1}	15	1	M∧M∧H	{0.4 0.5 0.1}
2	1	L∧L∧M	{0 0.1 0.9}	16	1	M∧H∧L	{0.4 0.6 0}
3	1	L∧L∧H	{0.1 0.1 0.8}	17	1	M∧H∧M	{0.5 0.3 0.2}
4	1	L∧M∧L	{0.1 0.2 0.7}	18	1	M∧H∧H	{0.5 0.4 0.1}
5	1	L∧M∧M	{0.1 0.3 0.6}	19	1	H∧L∧L	{0.4 0.3 0.3}
6	1	L∧M∧H	{0 0.4 0.6}	20	1	H∧L∧M	{0.5 0.3 0.2}
7	1	L∧H∧L	{0.1 0.4 0.5}	21	1	H∧L∧H	{0.6 0.2 0.2}
8	1	L∧H∧M	{0.2 0.2 0.6}	22	1	H∧M∧L	{0.6 0.3 0.1}
9	1	L∧H∧H	{0.2 0.3 0.5}	23	1	H∧M∧M	{0.6 0.2 0.2}
10	1	M∧L∧L	{0 0.5 0.5}	24	1	H∧M∧H	{0.7 0.2 0.1}
11	1	M∧L∧M	{0.1 0.6 0.3}	25	1	H∧H∧L	{0.8 0.2 0}
12	1	M∧L∧H	{0.2 0.5 0.3}	26	1	H∧H∧M	{0.9 0.1 0}
13	1	M∧M∧L	{0.2 0.6 0.2}	27	1	H∧H∧H	{1 0 0}
14	1	M∧M∧M	{0.3 0.6 0.1}				

**Table 3:** Initial parameters of the nonlinear membership function.

$s_{ij}$	j=1	j=2
i=1	0.5	0.7
i=2	2	1.5
i=3	1	2

**Table 4:** The rules of optimized BRB.

No.	$\theta_{l}$	$D_{0}\wedge D_{1}\wedge D_{2}$	Consequent {B,M,S}	No.	$\theta_{l}$	$D_{0}\wedge D_{1}\wedge D_{2}$	Consequent {B,M,S}
1	0.81	L∧L∧L	{0.13 0.06 0.81}	15	0.80	M∧M∧H	{0.44 0.46 0.10}
2	0.91	L∧L∧M	{0.13 0.16 0.71}	16	0.14	M∧H∧L	{0.35 0.61 0.04}
3	0.13	L∧L∧H	{0.10 0.11 0.79}	17	0.42	M∧H∧M	{0.41 0.33 0.26}
4	0.91	L∧M∧L	{0.20 0.25 0.55}	18	0.92	M∧H∧H	{0.54 0.34 0.12}
5	0.63	L∧M∧M	{0.18 0.25 0.57}	19	0.79	H∧L∧L	{0.39 0.28 0.33}
6	0.10	L∧M∧H	{0.02 0.41 0.58}	20	0.74	H∧L∧M	{0.52 0.29 0.19}
7	0.28	L∧H∧L	{0.13 0.34 0.54}	21	0.66	H∧L∧H	{0.51 0.23 0.27}
8	0.55	L∧H∧M	{0.26 0.19 0.55}	22	0.34	H∧M∧L	{0.50 0.36 0.14}
9	0.96	L∧H∧H	{0.27 0.32 0.41}	23	0.85	H∧M∧M	{0.57 0.26 0.18}
10	0.84	M∧L∧L	{0.13 0.43 0.43}	24	0.93	H∧M∧H	{0.69 0.20 0.12}
11	0.16	M∧L∧M	{0.10 0.52 0.38}	25	0.68	H∧H∧L	{0.67 0.24 0.09}
12	0.93	M∧L∧H	{0.25 0.44 0.31}	26	0.76	H∧H∧M	{0.76 0.17 0.07}
13	0.45	M∧M∧L	{0.30 0.46 0.24}	27	0.74	H∧H∧H	{0.92 0.03 0.06}
14	0.49	M∧M∧M	{0.33 0.57 0.11}				

**Table 5:** Optimized parameters of the nonlinear membership function.

$s_{ij}$	j=1	j=2
i=1	0.65	0.84
i=2	2.12	1.76
i=3	1.05	2.41

**Table 6:** Comparison of modeling accuracy of different BRBs.

	Initial BRB	BRB-n	BRB-g	BRB-t
Diagnostic accuracy	65.68%	**95.56%**	90.74%	87.21%

**Table 7:** Comparison of modeling accuracy of data-driven models.

	BRB-n	RF	NB	KNN
Diagnostic accuracy	**95.56%**	84.72%	76.30%	72.91%

**Table 8:** Comparison of modeling accuracy of different optimization algorithms.

	BRB-DE	BRB-PSO	BRB-PCMAES	BRB-GB
Diagnostic accuracy	88.59%	89.41%	92.74%	**95.56%**

**Table 9:** The distribution of belief degree of the first rule in the four BRBs.

Model	Belief Degree of the First Rule	The Most Supported Grade
Initial BRB	{0 0 **1**}	S
BRB-GB	{0.13 0.06 **0.81**}	S
BRB-DE	{0.21 **0.52** 0.27}	M
BRB-PSO	{0.15 **0.65** 0.20}	M
BRB-PCMAES	{**0.43** 0.28 0.29}	B

## Data Availability

Not applicable.
